# Mistletoe and Immunomodulation: Insights and Implications for Anticancer Therapies

**DOI:** 10.1155/2019/5893017

**Published:** 2019-04-17

**Authors:** Shiao Li Oei, Anja Thronicke, Friedemann Schad

**Affiliations:** ^1^Research Institute Havelhöhe, 14089 Berlin, Germany; ^2^Oncological Centre, Hospital Havelhöhe, 14089 Berlin, Germany

## Abstract

In early tumor development, cancer cells develop a plethora of strategies to escape surveillance from the adaptive and innate immune system. Cancer immunotherapies, in particular immune checkpoint inhibitors, are becoming a highly promising cancer therapeutic approach that has remarkable increased progress in combating various cancer types. Unfortunately, their mechanisms of action induce some complications, such as inflammatory reactions and immune-related adverse events. In the management of side effects during anticancer therapy, complementary and integrative therapy approaches are becoming of growing interest. Particularly, mistletoe,* Viscum album* L. (VA), has a long traditional history of about 100 years as an add-on therapy of cancer treatment in German-speaking countries. Besides antitumoral and quality of life-promoting activities, VA applications reduce side effects of modern conventional anticancer therapies and exert immunomodulatory characteristics. As these properties may provide a good basis for a combination with modern oncological therapies, the biological activities of VA applications and mechanisms involved have to be understood. In this review, the impact of VA compounds on different cellular pathways and immunological reactions in the fight against cancerous cells is discussed.

## 1. Cancer Immunotherapy

Cancer cells are able to gain control over a number of inhibitory pathways that are important for controlling immune responses and a major challenge of cancer therapy is to overcome immune resistance promoting tumor survival [1, 2]. The interplay between tumors and the immune system has long been known to involve complex interactions between tumor cells, immune cells, and the tumor microenvironment. For example, gain of expression of immunoinhibitory molecules such as programmed cell death protein 1 (PD-1) or altered expression of components involved in apoptosis is leading to apoptotic resistance. Considering different signaling pathways and strategies to overcome immunosuppression and to enhance the immunogenicity of tumors (by reverting their immune escape), various immunotherapies have been developed. A very promising approach led recently to the design of immune checkpoint inhibitors (ICIs), which in the meantime have increasingly been studied and used as a successful therapy for the treatment of various tumor types [3]. The immune checkpoint proteins cytotoxic T-lymphocyte-associated-4 (CTLA-4) and PD-1 are receptors, expressed on the surface of cytotoxic T-cells, which interact with their specific ligands (CD80/CD86 and PD-L1, resp.). These pathways can be exploited by cancer cells to escape from T-cell-mediated cell death [4]. Despite the significant benefits of ICIs, these drugs affect multiple organ systems, and their use can be associated with immune-related adverse events such as inflammatory arthritis, myositis, vasculitis, alveolitis, and further syndromes, which require appropriate long-term management [5]. Generally, clinical efficacy of anticancer therapy often is accompanied by side effects that affect the patient's quality of life and can lead to treatment discontinuations. Therefore, there is a considerable interest for supportive therapies counteracting toxicities without interfering with the elimination of cancerous cells. Another strategy to stimulate T-cells against tumor-specific epitopes is the development of therapeutic vaccines. These interventions include the identification of appropriate tumor antigens as targets for therapy [6]. The specificity of therapeutic vaccination combined with immunomodulation offers an attractive avenue for the development of cancer therapies. In comparison with clinical results of ICIs, vaccines have been less impressive; nevertheless, some combinations of ICIs with vaccines treatment seem to be promising for certain cancer diseases [7]. Furthermore, in most recent studies with oncolytic viruses combined with ICI therapy, it was suggested that antiviral immunological events may inflame the tumor and make it “hot” and suitable for subsequent ICI treatment [8]. Thus, combinational strategies could present an attractive opportunity for fighting cancer in the future.

## 2. Viscum Album Extracts and Immunomodulation

Mistletoe,* Viscum Album *L. (VA), therapy as an add-on therapy is among the most frequently used integrative oncological drugs in several countries in Europe [24]. Aims of add-on VA therapy are the improvement of health-related quality of life and the reduction of adverse events (AEs) associated with conventional anticancer strategies and, additionally, various immunomodulating activities have been described [25, 26]. The elucidation of immunostimulatory mechanisms of VA preparations and further validations in the context of clinical studies are critical in understanding the importance of add-on VA therapy. VA extracts contain a variety of compounds including mistletoe lectins and viscotoxins which have been ascribed to exert immunomodulatory effects [27]. Here, we discuss the impact of VA extracts between the immune system and cancer. Considering the abundance of published findings on this topic, we focus on preclinical and clinical findings in the context of primary effects of VA extracts on human immunological pathways. In a clinical study with 43 healthy volunteers, it was observed that, in an immediate response to a subcutaneous VA application, the numbers of leukocytes, granulocytes, and eosinophil cells increased [9]. Furthermore, the impact of VA extracts on the functionality of T-lymphocytes was studied. In a placebo-controlled trial with 71 healthy subjects, it was observed that subcutaneous applications of VA extracts resulted in eosinophilia and an increase of CD4 T-lymphocytes [10]. In addition, the specific immune system is also activated, as evident from the production of specific antibodies raised against VA lectins and viscotoxins, demonstrated in a randomized controlled trial with 47 healthy volunteers [11]. In a clinical study with eight cancer patients after application of VA extracts, the cytokine levels in serum increased [12]. In another clinical study with 10 breast cancer patients, after subcutaneous injections of VA lectins, a stimulation of natural killer (NK) and T-helper cells was detected [14]. Immunization experiments with mice showed that lectins are potent immunoadjuvants to enhance cellular and humoral immune responses [18]. In a study with cultured tumor cells, Korean mistletoe lectins exhibited immunomodulatory properties by enhancing dendritic cell maturation [22]. A maturation-inducing effect on human dendritic cells by application of VA extracts was shown in an* in vitro* study using a human cellular system [23].

After administration of VA extracts to tumor patients, the numbers of lymphocytes and NK cells increased [13]. In cultured glioblastoma cells, VA extracts inhibited tumor growth and enforced NK cell-mediated lysis of glioblastomas [16]. Several immunomodulatory effects in response to a single intravenous infusion of VA extracts such as neutrophilia, enhancement of phagocytic activity of granulocytes, and increase of NK cells have been reported [15]. NK cells play an important role in antitumoral immunity as they mediate the elimination of tumor cells and regulate the adaptive immunity. Cell experimental analyses revealed that viscotoxins are responsible for the increase in NK cell-mediated cytotoxicity [17]. In a clinical study of 70 cancer patients, it was observed that perioperative VA applications during digestive surgery increased the number of NK cells, in particular T-helper cell counts [19], and in a study with 98 breast cancer patients, a prevention of surgery-induced inhibition of the oxidative burst in granulocytes by intravenous application of VA extracts was shown [20]. A clinical trial with 62 colorectal cancer patients revealed that VA extracts can prevent suppression of NK cell activities [21]. A summary of the immunological activities of VA applications, which have been derived from preclinical and clinical studies, is given in Table 1.

Against this backdrop, complex immunological cross-talks and synergistic interactions of VA-mediated pathways may be critical for the entire process to eliminate cancerous cells. Due to the complexity of tumor development, it can be expected that addressing different pathways by using combinational treatment procedures may be a promising approach. Modern immunotherapies in combination with add-on VA could therefore be a helpful strategy to achieve synergism in cancer treatment efficacy. One of several well-studied immunological signaling pathways in which VA is involved is outlined below.

## 3. VA and the Cyclooxygenase Signaling Pathway

Bioactive phytochemicals are able to act as natural immunomodulators [28]. In this regard, a recent review reported that phytochemicals such as polyphenolic substances of green tea may exhibit anti-inflammatory and anticancer effects [29]. For inflammatory reactions, the cyclooxygenases (COX) are critical enzymes for several pathways including cytokine-induced secretion of prostaglandin E_2_. Some VA preparations were reported to have inflammatory properties and preclinical studies indicated that certain phytochemicals including components derived from VA preparations reduce selectively COX-2 levels [30–32]. COX-1 is constitutively and stably expressed at low levels in many tissues and involved in gastrointestinal, renal, vascular, and other physiological functions. As a result of a cross-talk between several mediators of inflammation, such as interleukins and cytokines, COX-2 is highly induced and its upregulation correlates with a poor cancer prognosis [33]. The COX-2 activity can be regulated at different levels. Various corticosteroids are available to suppress the immune system and reduce inflammatory reactions, but their use can be associated with a variety of side effects [34]. Traditional nonsteroid anti-inflammatory drugs (NSAID) inhibit the enzymatic activity of all COX enzymes and this also produces gastrointestinal toxicity, due to the inhibition of production and secretion of physiological prostaglandins. In difference to treatment of occurring AEs with NSAIDs, modern selective COX-2 inhibitors are efficient and showed a better safety profile than do nonselective NSAIDs, but adverse cardiovascular events may occur [35]. When COX-2 inhibitors have to be withdrawn, typically due to the occurrence of cardiovascular side effects, disease-modifying antirheumatic drugs are used to arrest progression of inflammatory reactions and relief from pain; however, these antirheumatics also inhibit other important immunological reactions. Hence, there is a need for anti-inflammatory agents, targeting the control of COX-2 levels but balancing safety and efficacy. Interestingly, for some phytotherapeutics, including VA preparations, it was shown that they influence COX-2 activity. Therefore, it was suspected that these phytochemicals may exert an anti-inflammatory effect via this pathway [29, 36]. It was shown that VA preparations reduced selectively COX-2 levels [30] and further experiments confirmed that VA preparations have the capacity to downregulate induced COX-2 activities by posttranscriptional destabilization of its transcripts [32]. From a retrospective analysis of medical records of 324 colorectal cancer patients, it was suggested that this proposed VA-mediated mechanism may contribute to alleviating inflammatory activities involved in cancer-related fatigue [37]. In difference to unspecific downregulation of COX activities by corticosteroids or NSAIDs, this proposed VA-dependent anti-inflammatory mechanism does not interfere with physiological functions, since COX-1 activity is not affected. It was hypothesized that this supportive VA-associated activity may contribute to various beneficial effects observed in clinical studies [30, 38].

## 4. Cancer, Immunity, and Safety Concerns

Numerous efforts have been undertaken to develop ways of stimulating the cellular immune response to eradicate tumors. The progress of checkpoint inhibitors in the clinical setting in the last decade has highlighted again the role of the immune system in the fight against cancer. Immune checkpoint therapy has revolutionized cancer treatment and has fundamentally changed the outcome for certain groups of patients with advanced cancer [3]. However, the application of ICIs is associated with relevant side effects, rarely even with lethal consequences [39, 40]. Usual treatments of drug-related AEs are therapy disruption and/or the application of corticosteroids or other immunosuppressant agents [41], which can reduce the signs and symptoms of inflammatory conditions but also have a general suppressing effect on the whole immune system, thus possibly interfering with the body's own defenses against tumor cells. The drug-related AEs of third-generation ICIs are lowered in relation to early developed inhibitors but still may cause severe immune responses, which can occur with a time delay and therefore cannot always be clearly assigned [39, 41]. Moreover, combinations of different ICIs seemed to increase grade 3-4 AEs in cancer patients [42] and also potentiation in toxicities has been seen with ICIs in combination with chemotherapy [39, 43]. Therefore, research and development of treatment strategies to optimize clinical positive outcomes and minimize occurring AEs of cancer patients is a key challenge for the future.

The cytotoxic and immunomodulatory properties of different VA preparations and applications have been intensively studied in the last years. Add-on VA therapy has been reported to entail a sound safety profile with no serious side effects. VA-associated AEs were reported but appeared to be dose-dependent and primarily confined to reactions at injection site and mild, transient pyrexia and flu-like symptoms [44–47]. Only in rare cases under intravenous VA treatment in a dose-dependent fashion were pseudoallergic hypersensitivity reactions described [48]. Hence, the application of VA extracts exhibits only low risks and seems to be safe but should be monitored by clinicians when applied in high dosages.

VA preparations, which have been shown to reduce AEs of chemo- and radiotherapy in cancer patients [26], may also exert positive effects during targeted therapy. In a randomized phase II study with 72 advanced lung cancer patients, it was observed that chemotherapy dose reductions, grade 3-4 nonhaematological side effects, and hospitalizations occurred less frequently in patients treated with add-on VA [49]. Utilizing registry data of 310 cancer patients, in a recent observational study, we observed a significant reduction of AE-induced treatment discontinuations in cancer patients, when treated with VA applications in addition to targeted therapy [50]. Furthermore, previously we evaluated the clinical safety of ICIs with add-on VA in patients with advanced or metastatic cancer [51]. This pilot observational study indicated that the ICI-induced AE rate was not adversely influenced by concomitant VA therapy. Further observational analyses have suggested that the combined treatment with ICIs and VA might even lower the AE rate including the immune-related AE rate [52]; however, no final conclusion is to be drawn yet due to a small patient number. More clinical trials are needed to characterize VA-mediated reduction of AE rates and may elucidate whether a combined treatment of ICIs and VA may have synergistic effects for safety-relevant outcomes.

## 5. Clinical Relevance and Perspectives

With the increasing knowledge of molecular signaling pathways and pathological mechanisms involved in progression of cancers, collaborative strategies have to be elaborated to optimize therapeutical outcomes. There is growing evidence that VA substances can exert apoptotic and cytotoxic as well as anti-inflammatory and immunological effects during cancer therapies. Some AEs resulting from anticancer therapies are no life-threatening diseases; however, these AEs may severely affect patients' quality of life and even worse can lead to treatment discontinuations. A reduction of AEs would support the adherence to anticancer therapy and might concurrently improve clinical outcomes of these therapies. In a systematic review of 26 randomized controlled and further 10 nonrandomized trials, the influence of VA extracts on quality of life in cancer patients was evaluated [26]. All the nonrandomized and 22 of the randomized trials reported a benefit and the authors concluded that VA treatments seem to have an impact on quality of life and reduce side effects of conventional therapies such as chemotherapy and radiation [26]. In addition, significant better overall survival was reported in a randomized clinical trial of advanced pancreatic cancer patients treated with VA [53], and the beneficial impact on overall survival was further supported with two recent real-world observational studies of add-on VA-treated advanced or metastasized pancreatic cancer [54] and metastasized non-small cell lung cancer patients [55]. In conclusion, through balanced interactions in complex immunological pathways, VA may assist the elimination of cancerous cells and additionally supports standard oncological strategies by lowering adverse effects. This in turn can exert positive effects on quality of life and might improve tolerability of cancer treatments. An improved patient compliance in cancer treatments may lead to better clinical outcomes. Therefore, it might be promising to further examine how combinational immunomodulating strategies like add-on VA treatment are suited to reduce occurring AEs in immunooncology.

## Figures and Tables

**Table 1:** Immunological activities of applied VA extracts.

VA-mediated activities	Study type	References
Increase of leukocytes, eosinophils, and granulocytes	RCT, placebo-controlled trial	[9, 10]

Induction of specific antibodies against VA constituents	RCT	[11]

Increase of secretion of cytokines	Clinical study	[12]

Increase of lymphocytes	Clinical study	[13]

Increase of activity of natural killer (NK) cells	Clinical studies	[13–15]

Enforcement of NK-cell mediated lysis of glioblastomas	Preclinical study	[16]

Increase of neutrophils and increase of granulocyte activity	Clinical study	[15]

Increase of activity of NK cells	Preclinical study	[17]

Enhancement of cellular and humoral immune response	Preclinical study	[18]

Increase of the activities of NK cells during surgery	Clinical studies, RCT	[19–21]

Enhancement of dendritic cell maturation	Preclinical study	[22]

Abrogation of tumor-induced immunosuppression of dendritic cells	Preclinical study	[23]

NK: natural killer; RCT: randomized clinical trial; VA: *Viscum album* L.
